# The Preventive Effect of Decorin on Epidural Fibrosis and Epidural Adhesions After Laminectomy

**DOI:** 10.3389/fphar.2021.774316

**Published:** 2021-12-16

**Authors:** Qing Ding, Qi Wei, Gaohong Sheng, Shanxi Wang, Shaoze Jing, Tian Ma, Ruizhuo Zhang, Tianqi Wang, Wenkai Li, Xiangyu Tang, Hua Wu, Chaoxu Liu

**Affiliations:** ^1^ Department of Orthopedics, Tongji Hospital, Tongji Medical College, Huazhong University of Science and Technology, Wuhan, China; ^2^ Department of Medical Ultrasound, Tongji Hospital, Tongji Medical College, Huazhong University of Science and Technology, Wuhan, China; ^3^ Department of Orthopedics, Shanxi Bethune Hospital, Taiyuan, China; ^4^ Department of Radiology, Tongji Hospital, Tongji Medical College, Huazhong University of Science and Technology, Wuhan, China

**Keywords:** decorin, laminectomy, fibroblast, epidural fibrosis, epidural adhesion, Smad2/3

## Abstract

Laminectomy is commonly performed to treat degenerative spinal diseases by reducing compression on the spinal cord and nerve roots. The postoperative epidural fibrosis and epidural adhesions may result in failed back surgery syndrome, which is characterized by the symptoms of lower back pain or leg pain. There is currently no satisfactory treatment for this complication. The pathological processes of epidural fibrosis and epidural adhesions are relevant to the proliferation of fibroblasts, transdifferentiation of fibroblasts into myofibroblasts, and the excessive deposition of extracellular matrix (ECM) protein. According to reports, transforming growth factor-β1 (TGF-β1) played a vital role in the development of fibrosis by promoting aforementioned processes. Decorin, an endogenous proteoglycan and natural inhibitor of TGF-β1, has exhibited prominent anti-fibrosis activity in various scar formation and fibrosis models of many organs. However, the preventive effect of decorin on epidural fibrosis and epidural adhesions requires further investigation. Here, we investigated the therapeutic effects and potential mechanisms of decorin on epidural fibrosis and epidural adhesions. Our results indicated that decorin could significantly suppress the TGF-β1-induced proliferation, transdifferentiation, and extracellular matrix production in primary fibroblasts. Furthermore, Smad2/3 signaling pathway had been demonstrated to be involved in the preventive effect of decorin. Moreover, administration of decorin *in vivo* could notably inhibit epidural fibrosis and epidural adhesions after laminectomy. To date, there is no approved therapy to target TGF-β1 for the treatment of epidural fibrosis and epidural adhesions after laminectomy. Our research proved the anti-fibrosis effect of decorin, which may provide an effective and promising treatment for epidural fibrosis and epidural adhesions.

## Introduction

Laminectomy is a commonly performed surgical procedure to relieve compression on the spinal cord and nerve roots. Unfortunately, the operation is successful in some patients, but undesirable results may appear after laminectomy. According to previous studies, approximately 8–40% of patients suffer from failed back surgery syndrome (FBSS), which is characterized by chronic nerve root or lower back pain after laminectomy (Guyer et al., 2006; Chan and Peng, 2011). Extensive epidural fibrosis and epidural adhesions are considered to be important causes of FBSS (Rabb, 2010). Furthermore, treatment of this complication may lead to further issues such as nerve root injuries, dural tears, epidural bleeding, and infection (Yakovlev et al., 2014). For these reasons, it is of great clinical significance to prevent epidural fibrosis and epidural adhesions after laminectomy.

Although the underlying mechanism leading to epidural fibrosis is complex, the proliferation of fibroblasts and the deposition of excessive extracellular matrix (ECM) have been reported to play a predominant role in dural fibrosis and dural adhesions (Laurent et al., 2007). Many inflammatory cytokines and growth factors, such as interleukin (IL)-1, IL-6, and transforming growth factor-β1 (TGF-β1), will be produced at the laminectomy site, stimulating the proliferation of fibroblasts and ECM deposition (Sun et al., 2008; Braun and Diamond, 2014). According to previous studies, TGF-β1 plays an important role in the development of fibrosis by promoting the transdifferentiation of fibroblasts into myofibroblasts (Zhu et al., 2007; Mohan et al., 2010). Myofibroblasts are considered as chief perpetrators of fibrosis, and they are avid ECM synthesizers. Therefore, inhibition of TGF-β1 or TGF-β1-related pathways and the transdifferentiation of fibroblasts is a promising treatment to alleviate the progression of fibrosis.

Decorin, a small leucine-rich proteoglycan, can interact with various ECM proteins, cell growth factors, and cell surface receptors (Gubbiotti et al., 2016). The typical structural function of decorin is to adjust the diameter and space between collagen fibers in fibrous tissues, which are primarily composed of type I collagen (Han et al., 2019). Moreover, decorin is an endogenous substance in the ECM and a natural inhibitor of TGF-β1 (Border et al., 1992; Hocking et al., 1998; Zhu et al., 2007; Schneider et al., 2021). Furthermore, the levels of decorin are downregulated in fibrotic conditions such as a postburn hypertrophic scar, whereas TGF-β1 is increased compared with normal conditions (Zhang et al., 2007; Honardoust et al., 2011). We hypothesized that decreased decorin might contribute to the pathogenesis of epidural fibrosis and epidural adhesions. A variety of researches employing recombinant decorin or decorin gene therapy have demonstrated that decorin has anti-fibrosis activity in scar formation or fibrosis models in many organs (Li et al., 2004; Jang et al., 2016; Chouhan et al., 2019; Cianfarani et al., 2019; Jiang et al., 2020). Chouhan et al. (2019) synthesized a hydrogel that can sustain the release of decorin and significantly inhibit corneal scar formation. The research results of Li et al. (Li et al., 2004) indicated that decorin could exhibit remarkable anti-fibrosis effects in muscle tissue by inhibiting TGF-β1. In the research of Cianfarani et al. (2019), the ability of decorin to modify the recessive dystrophic epidermolysis bullosa process was studied by using a lentiviral system expressing human decorin. Overexpression of decorin improved survival rates and limited the development of finger contraction and paw deformity, which were tightly related to the decrease of TGF-β1 levels and activation of the TGF-β1 signaling pathway. In addition, our previous studies have shown that decorin could reduce the expression of myofibroblast markers in arthrofibrotic tissue and suppressed the initial process of arthrofibrosis *in vivo* (Tang et al., 2018). Thus, decorin may be an effective anti-fibrosis drug for treating epidural fibrosis and epidural adhesions.

In the present study, we focused on the preventive effect of decorin on epidural fibrosis and epidural adhesions after laminectomy. The influences of decorin on the proliferation and transdifferentiation of fibroblasts into myofibroblasts were studied *in vitro*, and molecular mechanisms were also investigated. Furthermore, a rat model of laminectomy was introduced to evaluate the anti-fibrosis effect of decorin.

## Materials and Methods

### Reagents

Recombinant human decorin (143-DE) and TGF-β1 (240-B) were procured from R&D Systems (Minneapolis, MN, USA). The inhibitor of Smad3 (SIS3) was supplied by MCE (New Jersey, USA). SIS3 is a pyrrolopyridine compound and can selectively block TGF-β1-dependent Smad3 phosphorylation and Smad3-mediated cellular pathway. It was stored as 5 mM solution in DMSO, and this solution was used after diluting with medium. Spongostan was purchased from XIANG’EN (XIANG’EN Medical Technology Development Co., Ltd., Nanchang, China). It was a sterile, water-insoluble, and absorbable hemostatic gelatin sponge. The spongostan was trimmed into 3 × 3 × 5 mm cubes for use as the biological scaffold. High glucose Dulbecco’s modified Eagle’s medium (DMEM) and fetal bovine serum (FBS) were acquired from Gibco (Grand Island, NY, USA). The antibodies specific for α-SMA (ab124964) and fibronectin (ab268020) were purchased from Abcam (Cambridge, UK). Antibodies against Collagen I (#72026), Smad2 (#5339), phosphorylated-Smad2 (p-Smad2) (#3108), Smad3 (#9253), p-Smad3 (#9520), Smad2/3 (#8685), and secondary antibodies (Anti-rabbit IgG, #7074 and Anti-mouse IgG, HRP-linked Antibody #7076) were obtained from Cell Signaling Technology (Danvers, MA, USA). Antibody against Collagen III (22734-1-AP) was procured from Proteintech Group (Wuhan, Hubei, China). Antibody specific for GAPDH (BM1623) was obtained from Boster (Wuhan, Hubei, China).

### Cell Culture and Passage

Human fibroblasts were acquired from the Zhong Qiao Xin Zhou Biotechnology (Shanghai, China). According to the protocol provided by the company, the cells were cultured in high glucose DMEM supplemented with 15% FBS and 1% penicillin/streptomycin solution in a 37°C, 5% CO_2_ incubator. The culture medium was refreshed every 3 days. After reaching 80–90% confluence, the cells were separated and passaged. Cells of passages 3–4 were used for subsequent experiments.

### Cell Viability and Proliferation

Cell Counting Kit-8 (CCK-8) assay was used to measure fibroblast viability and proliferation according to the manufacturer’s protocol (Beyotime, Nanjing, China). In brief, fibroblasts were inoculated into a 96-well plate at a density of 1 × 10^4^ cells/well. After adherence, cells were incubated with different concentrations of TGF-β1 (1, 2.5, 5, and 10 ng/ml) or treated with TGF-β1 (5 ng/ml) in combination with different concentrations of decorin (2.5, 5, and 7.5 µg/ml) for 48 h. Subsequently, 100 µl culture medium containing 10 μl CCK-8 solution was added into each well. Then, the plate was incubated for 1 h at 37°C in the dark. The absorbance of the cells at 450 nm in each well was detected by the spectrophotometric microplate reader (Bio-Rad, CA, USA).

### Quantitative Real-Time Polymerase Chain Reaction

In accordance with the manufacturer’s instructions, total ribonucleic acid (RNA) of the fibroblasts was extracted with an RNA isolation kit purchased from Omega (Guangzhou, Guangdong, China). Then, the concentration of RNA samples was detected by spectrophotometry at 260 nm (Bio-Rad, CA, USA). The ratio of A260/A280 was calculated to verify the purity of total RNA. The equal amounts of RNA samples (1 µg) were transcribed to synthesize cDNA using a cDNA synthesis kit (TOYOBO, Osaka, Japan). Afterward, the cDNA was amplified using SYBR Green Real-time PCR Master Mix (TOYOBO). The qPCR reaction was conducted at 95°C for 1 min followed by 39 cycles at 95°C for 15 s and 60°C for 15 s. The expression levels of the target genes were normalized to the corresponding GAPDH and were analyzed using the 2−ΔΔCq method.

### Immunofluorescence Staining

The fibroblasts were initially seeded into 24-well plates at a density of 2 × 10^4^ cells/well. Following stimulation with 5 ng/ml TGF-β1 or in combination with 7.5 µg/ml decorin, the cells were fixed in 4% paraformaldehyde for 30 min and permeabilized with 0.1% Triton X-100 for 5 min. Then, the cells were blocked with 5% normal goat serum for 1 h at room temperature. Subsequently, the cells were incubated with antibodies against collagen I, fibronectin, α-SMA, and Smad2/3 at 4°C overnight and probed with FITC-conjugated (green) anti-rabbit IgG antibody for 1 h. Then, the cells were stained with DAPI in PBS for 5 min. Finally, the fluorescence images of the cells were photographed using a fluorescence microscope (Evos Flauto; Life Technologies, USA).

### Western Blotting Analysis

After incubated with TGF-β1 (5 ng/ml) or in combination with decorin (5 and 7.5 µg/ml) for 48 h, the fibroblasts were lysed with RIPA Lysis Buffer (Boster, Wuhan, China) to extract whole-cell proteins. The BCA assay kit obtained from Boster (Wuhan, Hubei, China) was used to determine the protein concentration. Subsequently, equal amounts of each protein samples (25 µg) were separated by gel electrophoresis and transferred to PVDF membranes (Millipore, Billerica, USA). After blocking with 5% skimmed milk for 1 h, the membranes were then incubated with the specific antibodies against collagen I, collagen III, α-SMA, fibronectin, GAPDH, Smad2, p-Smad2, Smad3, and p-Smad3 (all 1:1,000) at 4°C overnight. The membranes were washed three times with TBST and incubated with correspondent secondary antibodies (1:10,000) for 1 h. The proteins were detected using a western electrochemiluminescence substrate kit (Thermo Pierce, MA, USA) and photographed using a Bio-Rad scanner system (CA, USA). The band intensity was quantified by Image Lab 5.1 software (Bio-Rad, CA, USA).

### Animals and Laminectomy Model

This study was approved by the Institutional Animal Care and Use Committee of Tongji Hospital of Huazhong University of Science and Technology. One hundred and twenty-eight male Sprague–Dawley rats (250–300 g) were purchased from the Laboratory Animal Center of our hospital. The animals were randomly split into four groups. Sham group (n = 32): accepted sham operation without laminectomy; model group (n = 32): only a laminectomy was performed; spongostan group (n = 32): a spongostan impregnated with 0.4 ml of saline solution was retained on the dura mater; decorin treatment group (n = 32): a spongostan impregnated with decorin (100 µg/kg) in 0.4 ml of saline solution was left on the dura mater. This dosage of decorin was selected based on earlier studies (Alan et al., 2011; Turkoglu et al., 2014). The rats were weighed and anesthetized by intraperitoneal injection of pentobarbital (4.0 mg/100 g weight). After the lower backs of the rats were shaved and sterilized, a midline incision of 2–3 cm was made with the first lumbar vertebra (L1) as the center to expose the bony posterior elements. Then, the L1 laminectomy was performed carefully to keep the dura mater intact and fully exposed. After satisfactory hemostasis, the topical agents were applied and the wounds were closed layer by layer. The rats were observed every day for 2 weeks after the operation to monitor their spinal nerve function and wound healing. At the 4 and 8 weeks after operation, half of the rats in each group were randomly selected for further study.

### Macroscopic Assessment

Macroscopic assessments of epidural adhesions were performed at 4 and 8 weeks after operation, respectively. Eight rats from each group were randomly selected and sacrificed using an overdose of anesthetics. The laminectomy site was explored again through the previous operative incision. To prevent deviation, the researchers were blinded to the animal information before exploring. The epidural adhesions were evaluated according to Rydell standard (Rydell, 1970) as follows: grade 0, epidural scar tissues were not adherent to the dura mater; grade 1, epidural scar tissues adhered to the dura mater, but easily dissected; grade 2, epidural scar tissues adhered to the dura mater dissected with difficulty without disrupting the dura mater; grade 3, epidural scar tissues adhered to the dura mater and could not be dissected.

### MRI Examination

MRI evaluations were conducted at 4 and 8 weeks postoperatively. The MRI examinations were performed with the Bruker 7.0T MR imaging system (Berlin, Germany). The axial and sagittal T2-weighted imaging sequences were carried out on the segment where the laminectomy was performed. The sets of MRIs were accomplished under the following conditions: repetition time, 3,000 ms; echo time, 33 ms; thickness, 0.8 mm; layers, 15; matrix size, 256 × 256. The epidural adhesions and epidural fibrosis in MRIs were evaluated by the radiologists independently. The classification of epidural fibrosis was analyzed based on the methods reported in previous literature (Ross et al., 1999; Wang et al., 2020). In short, the spinal canal was subdivided into four quadrants by drawing two vertical lines from the center of the dura sac. The two quadrants on the dorsal side of the dura sac were our observation areas. The degree of epidural fibrosis was graded according to grade 0–4: grade 0, no scars; grade 1, >0 to ≤25% of the quadrants were full of scars; grade 2, >25 to ≤50% of the quadrants were full of scars; grade 3, >50 to ≤75% of the quadrants were filled with scars; grade 4, 75–100% of the quadrants were full of scars.

### Histological Evaluation

Histological analyses were performed following the MRI scans at 4 and 8 weeks. The spine columns, including surrounding muscle tissue and epidural fibrotic tissue, were resected *en bloc* between the cephalad and caudal vertebral bodies with L1 as the center. The specimens were fixed with 4% paraformaldehyde for 48 h. After being decalcified with 10% EDTA, the samples were dehydrated in graded ethanol. Then, the spine columns were embedded in paraffin and axially cut into 5-µm-thick sections. H&E staining and Masson staining were both performed in the sections. Antibodies specific for collagen I and fibronectin were used for further immunohistochemical staining. The qualitative analyses of fibroblast infiltration were evaluated in H&E-stained sections. The following methods were used to score fibroblast infiltration: grade 1, <100 fibroblasts in each 400× field; grade 2, 100–150 fibroblasts in each 400× field; grade 3, >150 fibroblasts in each 400× field (Coskun et al., 2000). The dural thickness and epidural fibrosis grades were analyzed in Masson-stained sections. The thickness of the dura mater was evaluated by selecting three measurement points at the laminectomy site. The first measurement point was taken from the midpoint of the laminectomy site, and the second and third measurement points were selected at 2 mm from both sides of the first point (Gurer et al., 2015). The average values of the three measurement points were used for statistical analysis. Epidural fibrosis and epidural adhesions in Masson-stained sections were graded as follows: grade 0, there was no scar tissue around the dura mater; grade 1, only thin fibrous bands were observed between the scar tissue and the dura mater; grade 2, continuous adhesions were observed in less than two-thirds of the laminectomy defect; grade 3, scar tissue adhesions were large, affecting more than two-thirds of the laminectomy defect, or the adhesions extending to the nerve roots (He et al., 1995).

### Statistical Analysis

Values of the results were shown as mean ± SD. One-way ANOVA was applied for multiple comparisons, and the unpaired *t*-test was used to compare the statistical significance between the two groups. A *p* value <0.05 was considered statistically significant. Prism 8 software (GraphPad Software Inc., La Jolla, CA, USA) was used for statistical analysis in this work.

## Results

### Decorin Suppresses the TGF-β1-Induced Proliferation of Fibroblasts

**FIGURE 1:**
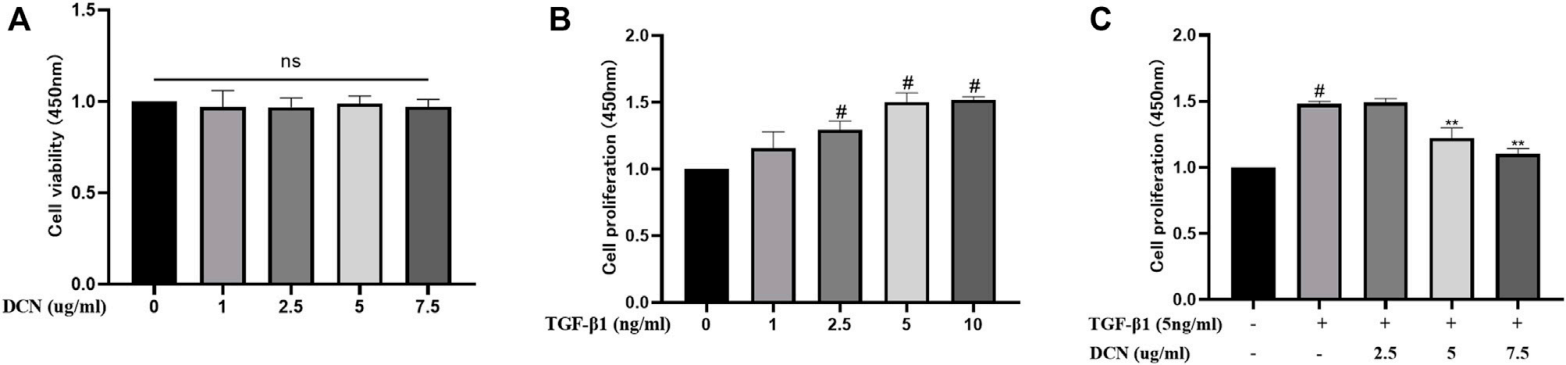
Effects of decorin (DCN) on cell viability and TGF-β1-induced proliferation are evaluated using a CCK-8 kit. The absorbance of the cells at 450 nm from each group is detected and recorded. **(A)** Fibroblasts were exposed to DCN (1, 2.5, 5, and 7.5 µg/ml) or **(B)** TGF-β1 (1, 2.5, 5, and 10 ng/ml) for 48 h. **(C)** Fibroblasts were treated with TGF-β1 (5 ng/ml) with or without DCN (2.5, 5, and 7.5 µg/ml) for 48 h. ns indicates no significance. ^#^ *p* < 0.05 vs control group; **p < 0.01 vs TGF-β1 group.

As shown in Figure 1A, decorin exhibited no cytotoxicity on fibroblasts at the concentrations of 1, 2.5, 5, and 7.5 µg/ml. To determine the proliferation of fibroblasts induced by TGF-β1, dose-response experiments with different concentrations of TGF-β1 (1, 2.5, 5, and 10 ng/ml) were conducted. As shown in Figure 1B, TGF-β1 promoted fibroblast proliferation in a dose-dependent manner. This promotion effect peaked when the concentration was 5 ng/ml. Following incubation with different concentrations of decorin (2.5, 5, and 7.5 µg/ml), the proliferation of fibroblasts induced by TGF-β1 (5 ng/ml) was notably suppressed by decorin with the concentrations of 5 and 7.5 µg/ml (Figure 1C). Decorin was used at doses of 5 and 7.5 µg/ml in subsequent studies.

### Effects of Decorin on TGF-β1-Induced Fibroblast Transdifferentiation

**FIGURE 2:**
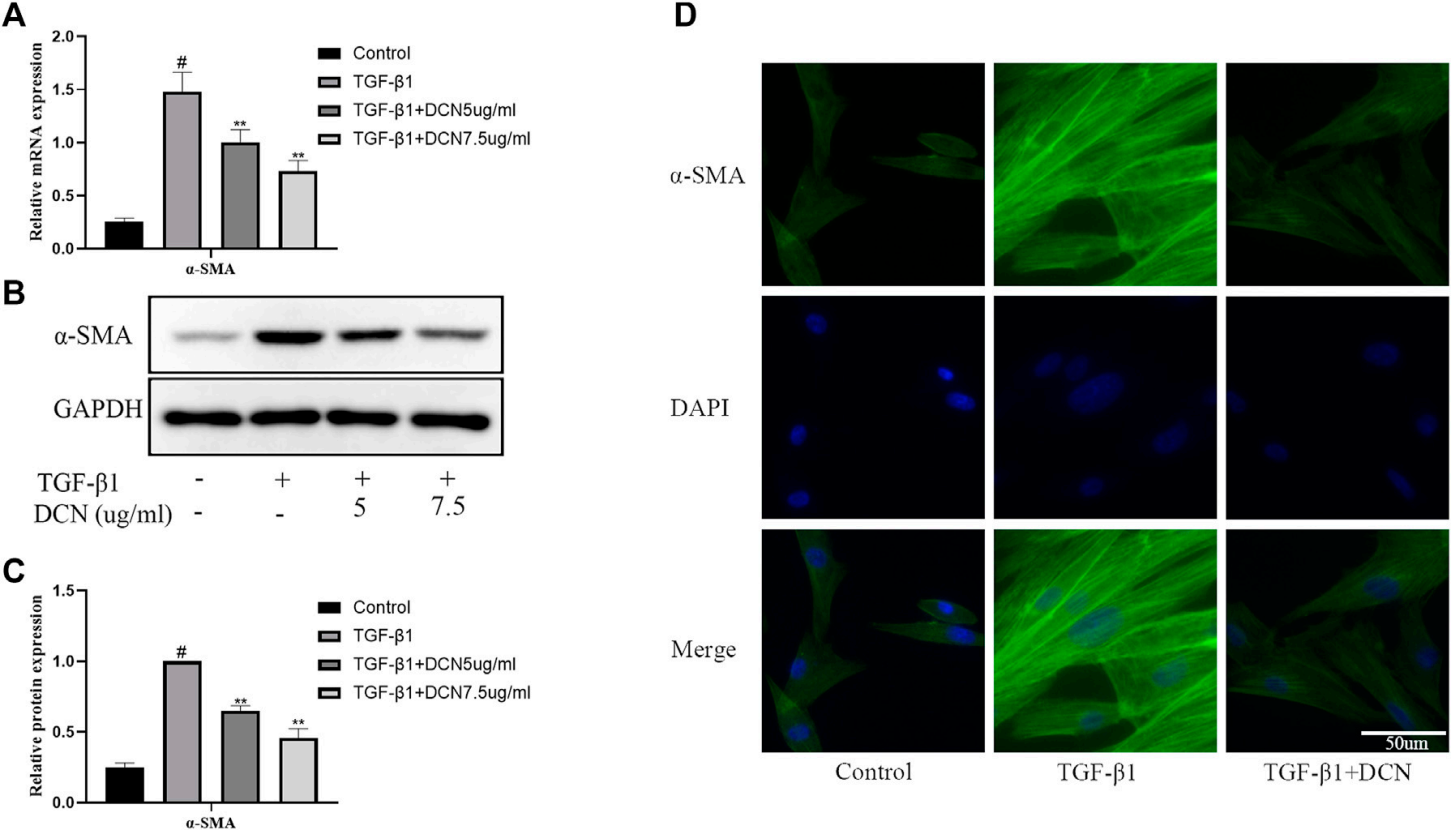
Decorin (DCN) suppresses TGF-β1-induced transdifferentiation of fibroblasts. Fibroblasts were exposed to TGF-β1 (5 ng/ml) with or without DCN (5 and 7.5 µg/ml) for 48 h. **(A)** Gene expression of α-SMA was detected by RT-qPCR. **(B)** Western blotting and **(C)** quantitative analysis of α-SMA in each group. **(D)** The α-SMA was observed by immunofluorescence after cells were treated with TGF-β1 (5 ng/ml) with or without DCN (7.5 µg/ml). ^#^ *p* < 0.05 vs control group; **p < 0.01 vs TGF-β1 group.

To investigate whether decorin could suppress transdifferentiation of fibroblasts into myofibroblasts induced by TGF-β1, the expression of α-SMA, a widely investigated myofibroblast marker, was detected by qPCR and western blotting. As exhibited in Figures 2A–C, the expression of α-SMA in fibroblasts induced by TGF-β1 was significantly increased, while decorin could significantly inhibit the increase of α-SMA in a dose-dependent manner. Furthermore, the immunofluorescence staining of α-SMA showed the same results (Figure 2D).

### Decorin Inhibits TGF-β1-Induced ECM Synthesis

**FIGURE 3:**
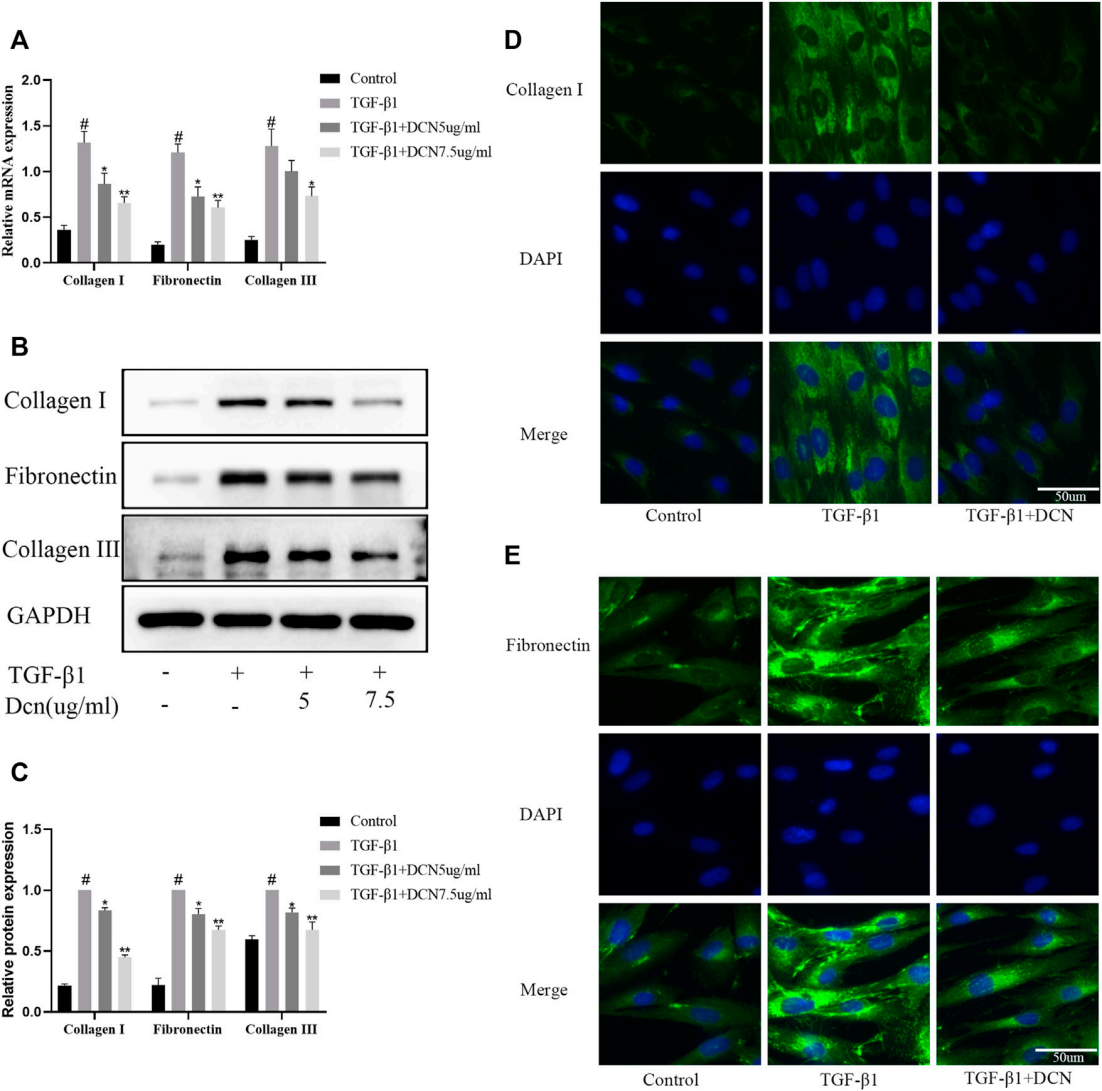
Decorin (DCN) inhibits TGF-β1-induced extracellular matrix synthesis. Fibroblasts were exposed to TGF-β1 (5 ng/ml) with or without DCN (5 and 7.5 µg/ml) for 48 h. **(A)** Gene expression of fibronectin, collagen I, and collagen III was detected by RT-qPCR. **(B)** Western blotting and **(C)** quantitative analysis of fibronectin, collagen I, and collagen III in each group. **(D)** Collagen I and **(E)** fibronectin were observed by immunofluorescence after cells were treated with TGF-β1 (5 ng/ml) with or without DCN (7.5 µg/ml). ^#^ *p* < 0.05 vs control group; *p < 0.05 vs TGF-β1 group; **p < 0.01 vs TGF-β1 group.

Collagen fibers and fibronectin are the major ECM components that lead to the formation of fibrosis. As exhibited in Figures 3A–C, TGF-β1 significantly induced the upregulation of fibronectin, collagen I, and collagen III. However, decorin could reverse these changes. The changes of fibronectin and collagen I were further confirmed by the fluorescent results (Figures 3D, E).

### Effects of Decorin on TGF-β1-Induced Smad2/3 Pathway Activation

**FIGURE 4:**
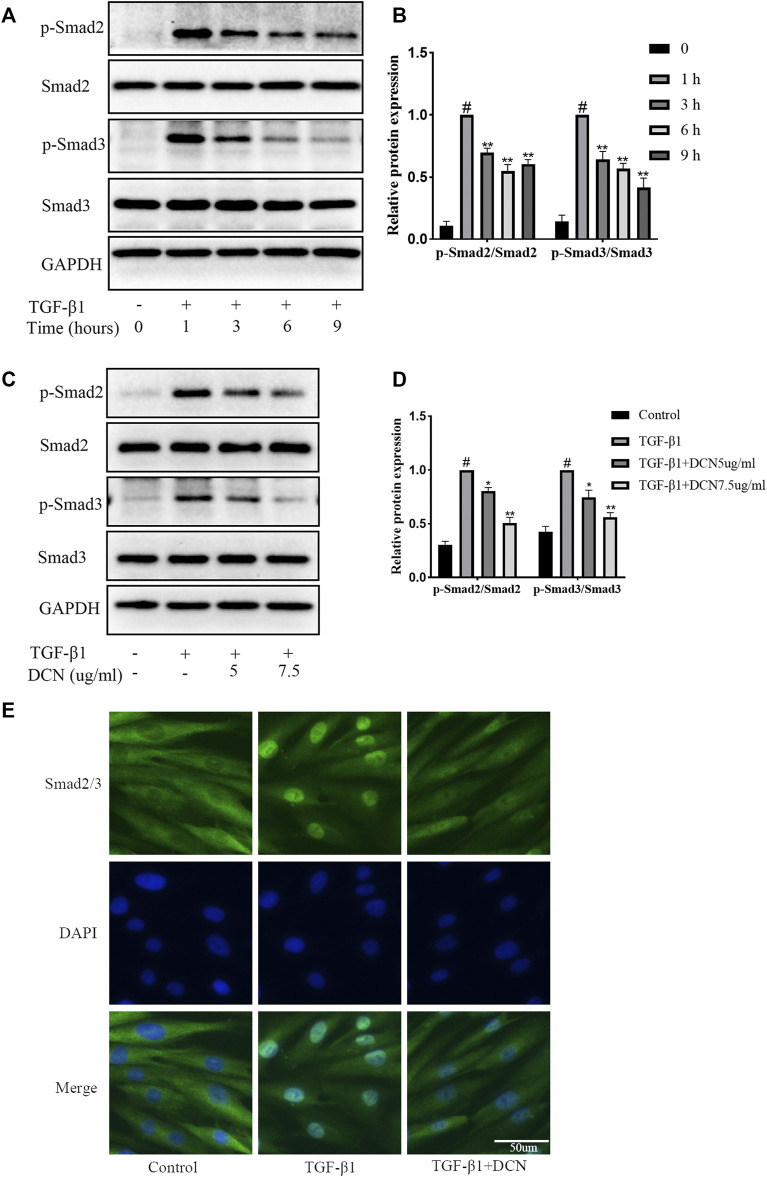
Decorin (DCN) inhibits TGF-β1-induced Smad2/3 signaling pathway activation. **(A)** Western blotting and **(B)** quantification analysis of Smad2, p-Smad2, Smad3, and p-Smad3 from TGF-β1-treated fibroblasts at different time points (0, 1, 3, 6, and 9 h). **(C)** Western blotting and **(D)** quantification analysis of Smad2, p-Smad2, Smad3, and p-Smad3 from fibroblasts after treated with DCN (5 and 7.5 µg/ml) for 2 h, followed by incubation with TGF-β1 (5 ng/ml) for 1 h. **(E)** Nuclear translocation of Smad2/3 was detected by immunofluorescence. ^#^ *p* < 0.05 vs control group; *p < 0.05 vs TGF-β1 group; **p < 0.01 vs TGF-β1 group.

The Smad2/3 signaling pathway is highly involved in the progression of fibrosis. Several time points (0, 1, 3, 6, and 9 h) were selected to verify the activation process after treatment with TGF-β1. The results in Figures 4A, B showed that activation of the smad2/3 signaling pathway induced by TGF-β1 was time dependent. The activation was the strongest after 1 h, and then gradually weakened. Therefore, we selected the activation peak after 1 h to explore the regulation of decorin on the smad2/3 signaling pathway. As exhibited in Figures 4C, D, TGF-β1 could markedly promote the activation of smad2 and smad3. However, when the fibroblasts were treated with decorin (5, 7.5 µg/ml), the phosphorylation levels of Smad2 and Smad3 were significantly reduced compared with TGF-β1 treated group. In addition, immunofluorescence staining was conducted to evaluate smad2/3 nuclear translocation. As exhibited in Figure 4E, smad2/3 was located in the cytoplasm area of unstimulated fibroblasts. Compared with the control group, we observed accumulation of smad2/3 in the nucleus of fibroblasts after stimulation with TGF-β1 for 1 h. However, this process could be partly repressed by decorin (7.5 µg/ml).

### TGF-β1-Induced Fibroblast Transdifferentiation and ECM Synthesis Were Inhibited by SIS3

**FIGURE 5:**
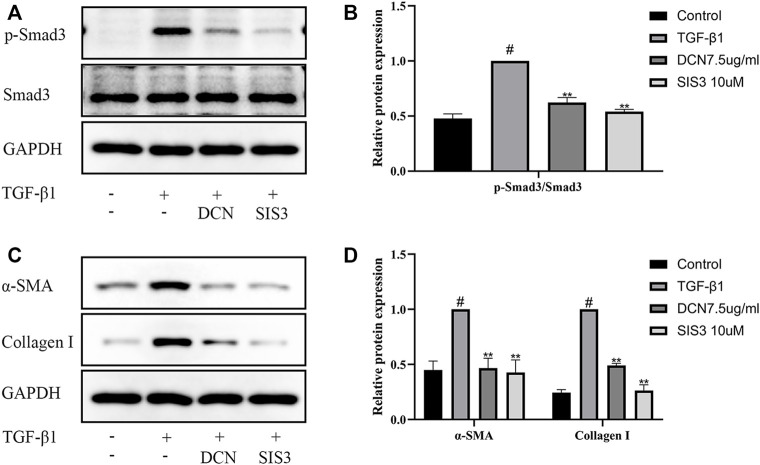
TGF-β1-induced Smad2/3 signaling pathway activation, fibroblast transdifferentiation, and ECM synthesis are inhibited by SIS3 (a novel specific inhibitor of Smad3). **(A)** Western blotting and **(B)** quantification analysis of Smad3 and p-Smad3 from fibroblasts after treated with DCN (7.5 µg/ml) or SIS3 (10 µM) for 2 h, followed by incubation with TGF-β1 (5 ng/ml) for 1 h. **(C)** Western blotting and **(D)** quantification analysis of α-SMA and collagen I from fibroblasts after treated with TGF-β1 (5 ng/ml) in the presence of DCN (7.5 µg/ml) or SIS3 (10 µM) for 48 h. ^#^ *p* < 0.05 vs control group; *p < 0.05 vs TGF-β1 group; **p < 0.01 vs TGF-β1 group.

To further investigate the role of the Smad2/3 signaling pathway in TGF-β1-induced fibroblast transdifferentiation and ECM synthesis, SIS3 (a novel specific inhibitor of Smad3) was used in this study. As expected, SIS3 could significantly inhibit the phosphorylation of Smad3 (Figures 5A, B). In addition, the induction of α-SMA and Collagen I by TGF-β1 was significantly inhibited by SIS3 (Figures 5C, D). These results were consistent with decorin (7.5 µg/ml) treatment, which indirectly suggested that decorin could at least partially inhibit TGF-β1-induced fibroblast transdifferentiation and ECM production via the Smad2/3 signaling pathway.

### Gross Observation of the Epidural Adhesions After Laminectomy

**FIGURE 6:**
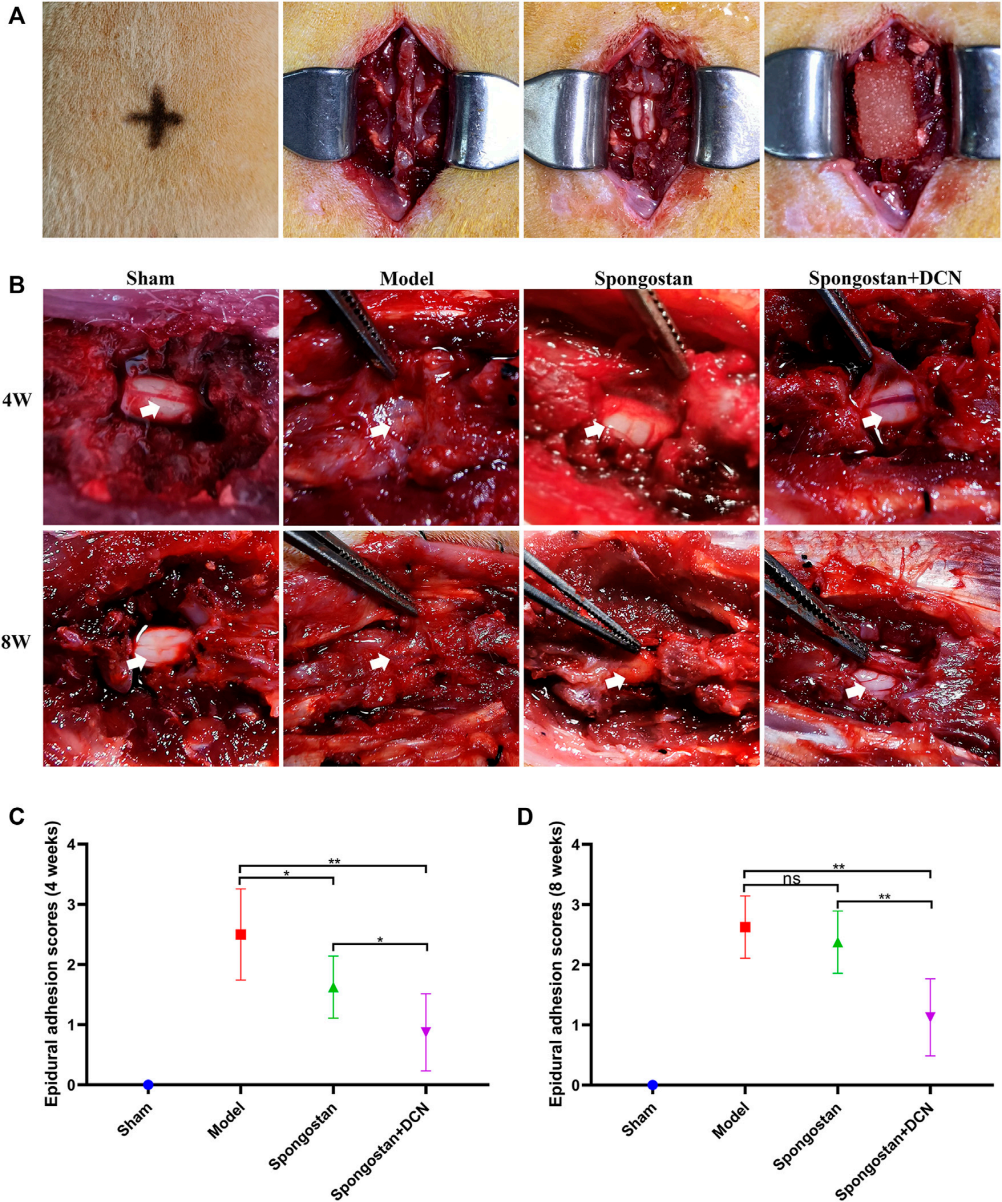
Establishment of a rat laminectomy model and gross observation of the epidural adhesions. **(A)** The process of building the model: surface location of L1, exposing the lamina, excision of lamina, and a spongostan impregnated with saline or decorin (DCN) solution was retained on the dura mater. **(B)** Gross observation of laminectomy sites after 4 and 8 weeks of treatment. White arrows indicate the dura mater. **(C)** Four weeks and **(D)** 8 weeks of epidural adhesion score results based on Rydell standard. ns indicates no significance. *p < 0.05; **p < 0.01.

To manifest the effects of decorin on the pathogenesis of epidural fibrosis and epidural adhesions *in vivo*, we established a rat laminectomy model. The process of building the model is shown in Figure 6A. Gross examinations of the wound sites did not reveal infection or poor wound healing. Gross observations of the epidural adhesions were carried out at the position of the original surgical incision. Representative macroscopic observation results of each group at 4 and 8 weeks are shown in Figure 6B. The dura mater in the sham operation group was smooth without adhesion. However, a large number of scar tissues were formed and adhered to the dura mater in model group, which were difficult to dissect and separate. In the spongostan group, the epidural scar tissues sticking with the dura mater were easy to dissect at 4 weeks. By the 8th week, the epidural scar tissues were difficult to dissect. By contrast, the decorin treatment group showed mild epidural adhesions, which can be bluntly separated by minimal manual traction without damaging the dura. Moreover, the surface of the dura mater was smooth after the removal of the epidural scar tissues. The epidural adhesion score was the lowest among all groups at 4 and 8 weeks, respectively (Figures 6C, D).

### MRI Evaluation

**FIGURE 7:**
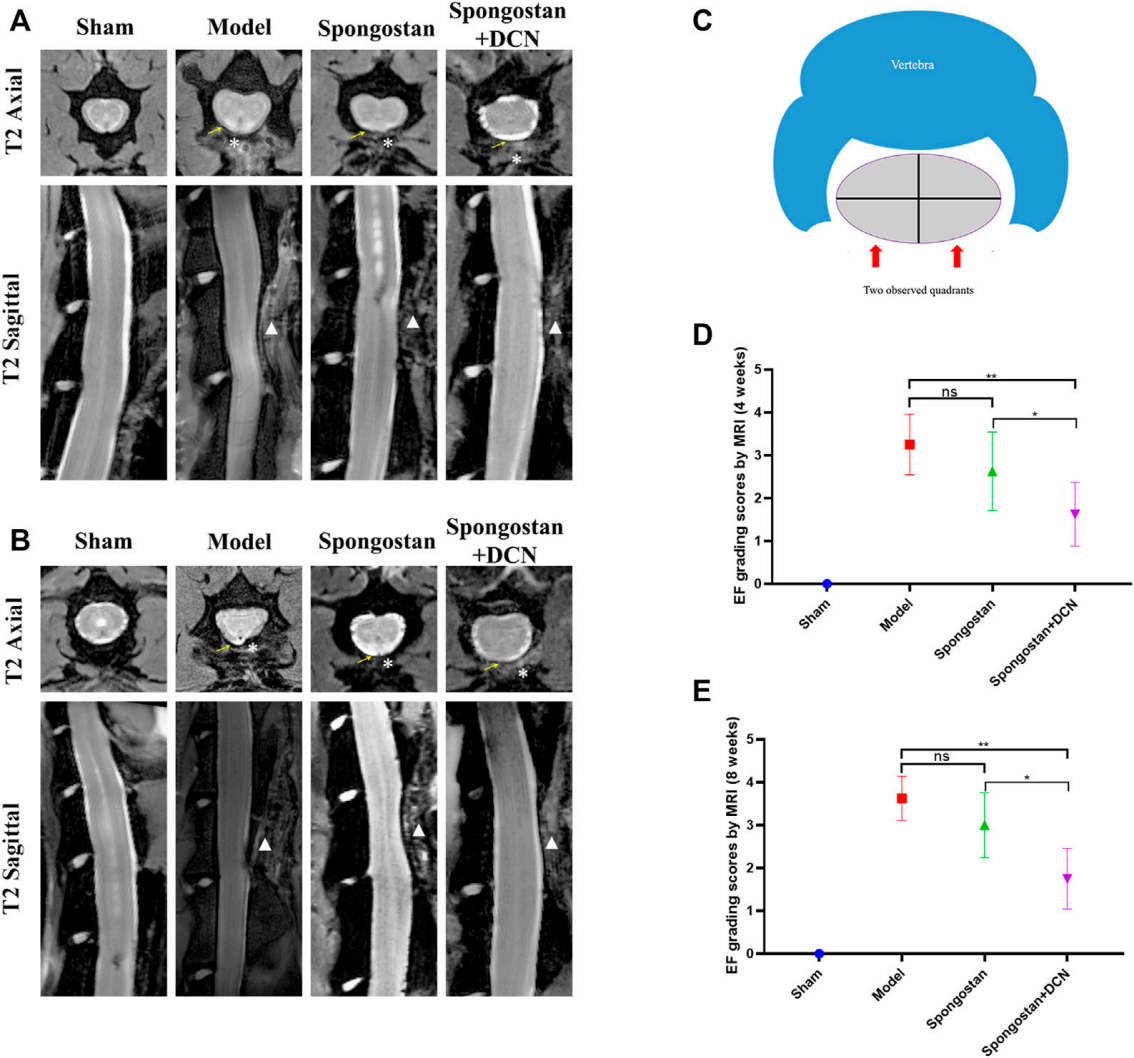
Results of MRI evaluation. The axial and sagittal T2-weighted MRIs of laminectomy sites at **(A)** 4 weeks and **(B)** 8 weeks. Yellow arrows indicate the dura mater. * indicates the epidural dense scar tissues. White triangles in sagittal images indicate lamina defect site. **(C)** Schematic diagram of the axial image used for epidural fibrosis (EF) scoring. **(D)** Four weeks and **(E)** 8 weeks EF score results based on MRIs. ns indicates no significance. *p < 0.05; **p < 0.01.

**FIGURE 8:**
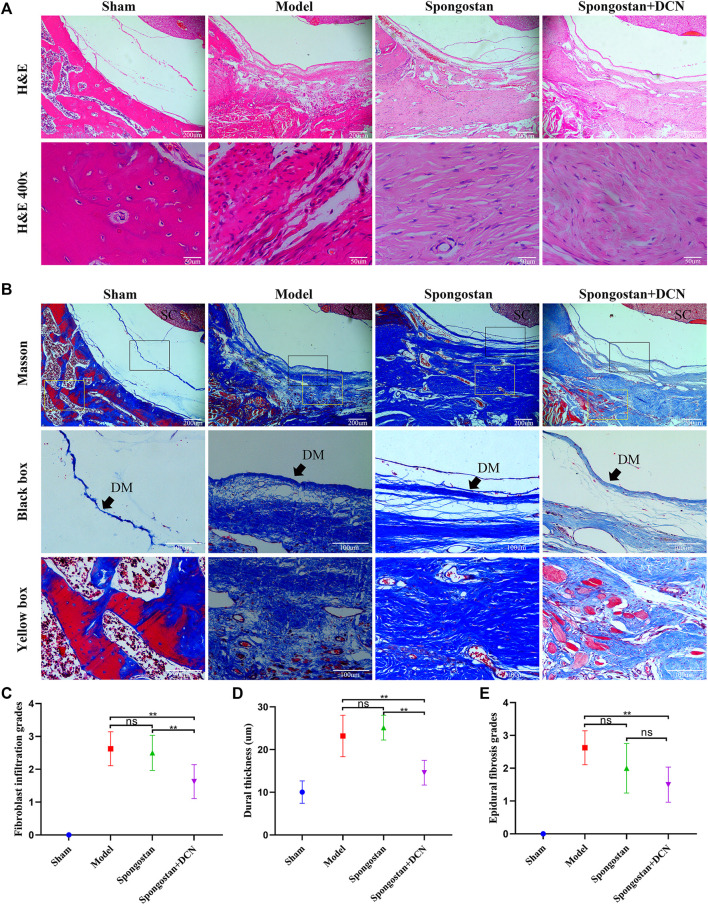
Representative H&E staining and Masson staining from each group at 4 weeks postoperatively. **(A)** H&E staining of spinal column in laminectomy sites. The high-resolution images (400×) in the bottom panel show fibroblast infiltration. **(B)** Masson-stained sections of laminectomy sites. DM, dura mater; SC, spinal cord. **(C)** Quantified fibroblast infiltration grades at high resolution (400×). **(D)** Comparison of dural thickness among groups. **(E)** Quantitative analysis of epidural fibrosis grades according to Masson-stained sections. *p < 0.05; **p < 0.01.

The axial and sagittal T2-weighted MRIs of the spinal column were obtained after 4 weeks (Figure 7A) and 8 weeks (Figure 7B) of treatment, respectively. In the sham group, the normal spinal structure of the rats could be observed. There was an intact lamina behind the dural sac and no compression on the dural sac. In contrast, the rats in the model group exhibited noticeable epidural scar adhesions and dense scar tissues compression on the dural sac. At the 4th week, a narrow gap between dense scar tissues and dura mater could be observed in the spongostan group. The dura mater was not obviously compressed. However, in the 8th week, the dense scar tissues increased significantly, causing compression on the dural sac. In the decorin treatment group, there was only a small amount of dense scar tissues around the dural sac at 4 and 8 weeks, and there was no compression on the dural sac. Consistently, similar results were found based on the grading scores of MRI (Figures 7C–E).

### Histological Analysis

**FIGURE 9:**
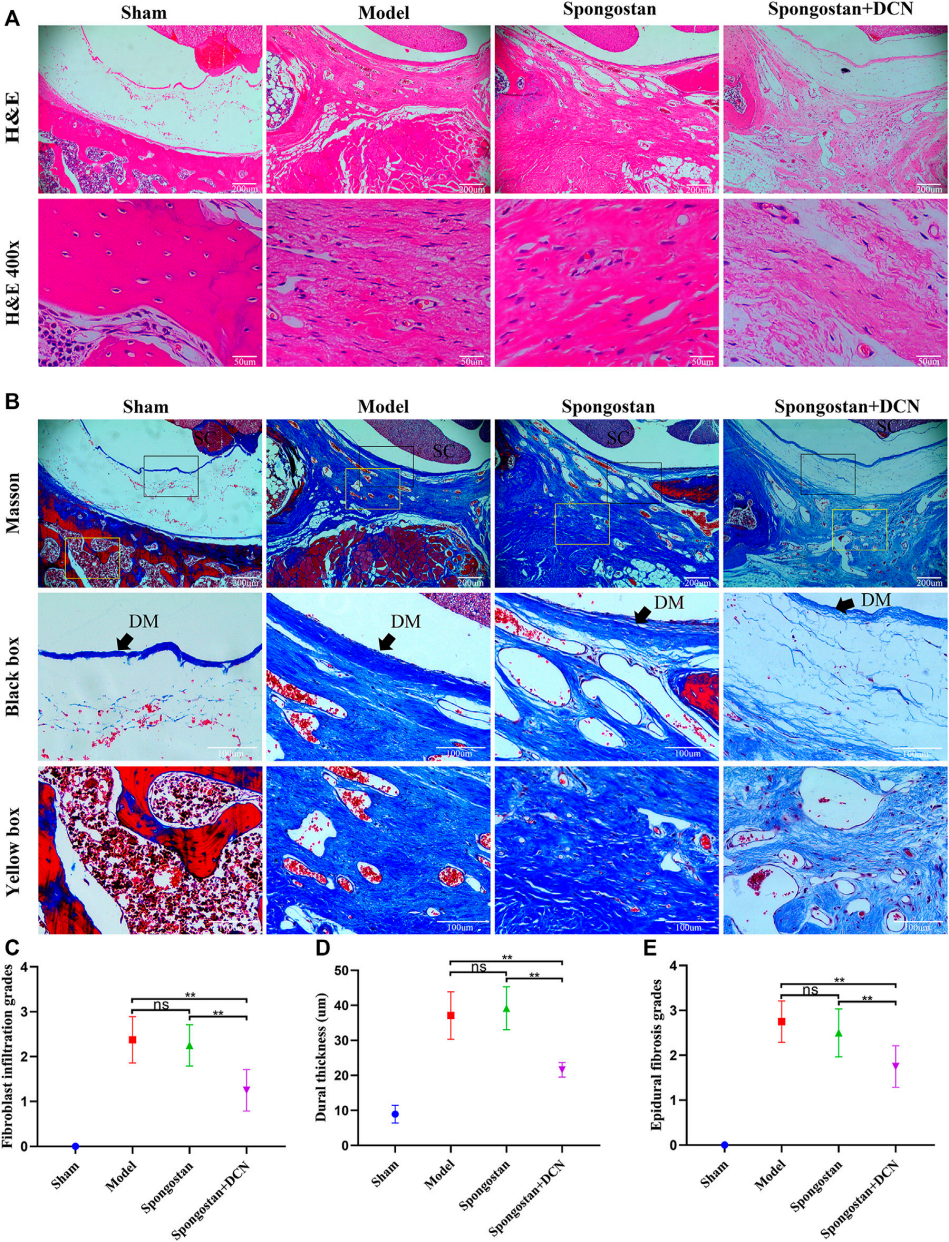
Representative H&E staining and Masson staining from each group at 8 weeks postoperatively. **(A)** H&E staining of spinal column in laminectomy sites. The high-resolution images (400×) in the bottom panel show fibroblast infiltration. **(B)** Masson-stained sections of laminectomy sites. DM, dura mater; SC, spinal cord. **(C)** Quantified fibroblast infiltration grades at high resolution (400×). **(D)** Comparison of dural thickness among groups. **(E)** Quantitative analysis of epidural fibrosis grades according to Masson-stained sections. *p < 0.05; **p < 0.01.

**FIGURE 10:**
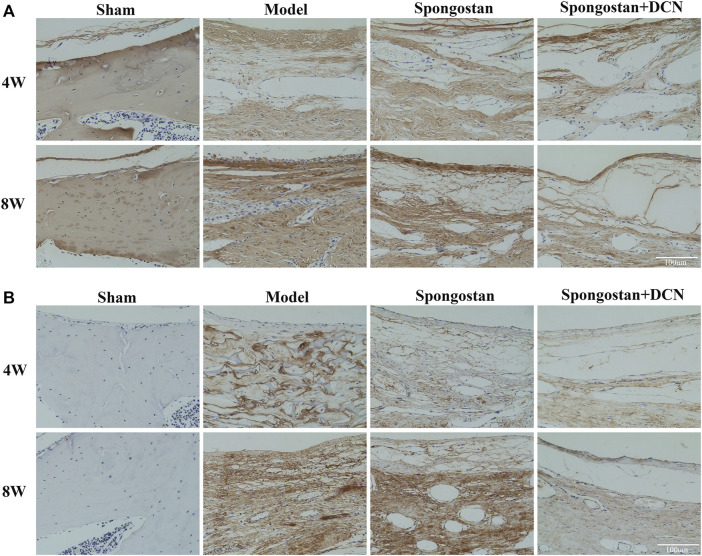
Decorin (DCN) inhibits the synthesis of collagen I and fibronectin *in vivo*. Immunohistochemical staining of collagen I **(A)** and fibronectin **(B)** from each group at 4 and 8 weeks, respectively.

Histological evaluations were also performed at 4 weeks (Figures 8A, B) and 8 weeks (Figures 9A, B). Notably, no epidural scar formation and epidural scar adhesions around the dura mater were observed in the sham group due to the barrier function of the intact lamina. In contrast, the outside dural canal was filled with abundant scar tissues, and the epidural adhesions were extensive and serious in the model group. In the spongostan group, although there was a fuzzy gap between dura mater and epidural scar tissues, it was filled by many fibrous connections at 8 weeks. By contrast, the gap in histological sections corresponding to the decorin treatment group was more evident at 4 and 8 weeks. In addition, the model and spongostan groups exhibited more abundant fibroblasts than the decorin treatment group. There were significant differences between the decorin and model group, and decorin and spongostan group (Figure 8C and Figure 9C). This indicated that decorin could inhibit the proliferation of fibroblasts *in vivo*. Furthermore, significantly higher mean thickness values of dura mater were observed in the model and spongostan groups than in the sham group at 4 and 8 weeks (Figure 8D and Figure 9D). There was no significant difference between the model and the spongostan group. However, there were significant differences in the decorin treatment group compared with the model and the spongostan group at 4 and 8 weeks. Accordingly, the lowest epidural fibrosis score was observed in the decorin treatment group, indicating significantly less epidural fibrosis and epidural adhesions in this group (Figure 8E and Figure 9E). The immunohistochemical staining results of collagen I (Figure 10A) and fibronectin (Figure 10B) showed a trend consistent with *in vitro* studies.

## Discussion

Epidural fibrosis and epidural adhesions are the common postoperative complications related to the spinal operation. The formation of epidural scar tissue causes compression on the dura mater and traction of nerve roots, which may result in FBSS. The existent therapeutic methods, including conservative treatment and surgical scar resection, are not satisfactory (Cruccu et al., 2007). Various methods have been explored to alleviate epidural fibrosis and epidural adhesions, such as topical or systemic treatment with drugs and the use of physical barriers based on biomaterials (Yan et al., 2013; Zeinalizadeh et al., 2014; Zhang et al., 2014; Gurer et al., 2015; Wang et al., 2020). However, only a few drugs and biomaterials have been included in clinical trials. There are almost no drugs to prevent epidural fibrosis and epidural adhesions after laminectomy. Obviously, epidural fibrosis and epidural adhesions are still a huge treatment challenge, and it is urgent to develop effective anti-fibrosis drugs. In this study, the anti-fibrosis effects of decorin were demonstrated *in vitro* using primary fibroblasts and further confirmed in the rat model after laminectomy.

The pathological processes of epidural fibrosis and epidural adhesions involved the proliferation, transdifferentiation of fibroblasts, and the excessive deposition of ECM protein. TGF-β1 was a valid fibrotic cytokine, which played a vital role in the process of fibrosis in many organs (Li et al., 2004; Luo et al., 2014; Xu et al., 2020; McArdle et al., 2021). In this research, the results suggested that exogenous TGF-β1 could promote the proliferation of fibroblasts in a dose-dependent manner, and this proliferative effect could be effectively inhibited by decorin. Moreover, it was reported that TGF-β1 could induce transdifferentiation of fibroblasts into myofibroblasts, which were characterized by the expression of α-SMA (Luo et al., 2014; Xu et al., 2020). We observed that stimulating fibroblasts with TGF-β1 induced a significant upregulation of α-SMA. After incubation with decorin, the upregulation of α-SMA induced by TGF-β1 was significantly suppressed. These changes were evident at both the transcription and translation levels. This indicated that decorin could repress the transdifferentiation of fibroblasts into myofibroblasts induced by TGF-β1. Furthermore, our results proved that decorin could inhibit the TGF-β1-induced synthesis of fibronectin, collagen I, and collagen III. Overall, these results demonstrated that decorin could inhibit the progress of epidural fibrosis. This was consistent with the previous results which demonstrated that the process of fibrosis can be alleviated by restraining the proliferation, transdifferentiation, and ECM production in TGF-β1-stimulated fibroblasts (Luo et al., 2014; Li et al., 2020; Xu et al., 2020).

TGF-β1 and its correlative signaling pathways are extensively implicated in the pathogenesis of fibrosis (Derynck and Zhang, 2003; Frangogiannis, 2020). In the classical pathway, the responses are triggered by the activation of TGF-β receptors. The active TGF-β receptor 1 then phosphorylates Smad2 and Smad3, which complex with Smad4 and translocate from the cytoplasm into the nucleus. After the complexes enter the nucleus, they bind to the targeted DNA and regulate gene expression (Derynck and Zhang, 2003). As reported in previous researches, the inhibition of Smad2/3 signaling pathway could significantly attenuate the formation of scar tissue in central nervous system and pulmonary fibrosis (Ahmed et al., 2014; Luo et al., 2014; Li et al., 2020). Therefore, in the present study, we focused on the pathway. We observed that the phosphorylation levels of Smad2 and Smad3 were significantly increased with TGF-β1 stimulation, and the translocation of Smad2/3 from the cytoplasm into the nucleus was confirmed with immunofluorescence staining. Decorin could evidently inhibit the aforementioned processes, which suggested that decorin could suppress the activation of the Smad2/3 pathway induced by TGF-β1. This was consistent with the anti-fibrosis mechanism of decorin described in previous studies (Zhu et al., 2007; Ahmed et al., 2014; Jang et al., 2016; Yan et al., 2016). In addition, the inhibition of Smad3 by SIS3 (an inhibitor of Smad3) markedly suppressed TGF-β1-induced fibroblast transdifferentiation and ECM synthesis, further emphasizing the importance of decorin in antagonizing the TGF-β1 signaling pathway.

To illustrate the therapeutic potential of decorin in epidural fibrosis and epidural adhesions, *in vitro* testing was far from enough. We further built a rat laminectomy model and assessed the preventive effect of decorin *in vivo*. First, we selected some rats for macroscopic evaluation of epidural adhesions. As expected, a large number of epidural scar tissues were formed and adhered to the dura mater in the model group, which were difficult to dissect and separate. In contrast, the decorin treatment group showed slight epidural adhesions that could be easily separated. Interestingly, the epidural scar tissues that adhered to the dura mater in the spongostan group were easy to peel off at 4 weeks. By the 8th week, the epidural scar tissues were difficult to dissect. This might be attributed to the physical barrier function of spongostan in the early stage. Similar results were shown in the subsequent MRI examinations. It was evident that the dura mater was compressed by the epidural scar tissues in the model group. In the decorin treatment group, the dura mater was not markedly depressed toward the spinal cord, indicating that epidural scars did not compress the dura mater. These results suggested that decorin had exerted significant anti-fibrosis activity *in vivo*. To further verify the anti-fibrosis effects of decorin and its mechanisms, histological analyses were carried out. Treatment with decorin significantly reduced fibroblast infiltration and collagen fiber deposition in epidural scar tissues. In addition, the immunohistochemistry staining showed that decorin could significantly reduce collagen I and fibronectin expression in epidural scar tissues. All data indicated that decorin could ameliorate epidural fibrosis and epidural adhesions after laminectomy.

However, this research is not without limitations. First, the most effective dose of decorin *in vivo* application is still unclear. Although its efficacy has been proven in this study, it is necessary to further evaluate the safety and effectiveness in large animals. Second, no sensation tests were performed to compare the sensory function of the animals before execution. We observed that the motor function of the animals was normal after different treatments. In future research, electrophysiological monitoring should be introduced to assess whether there is abnormal sensory function.

## Conclusion

In the present study, the anti-fibrosis effects of decorin were demonstrated *in vitro* and *in vivo*. We further proved decorin functioned by inhibiting TGF-β1-induced Smad2/3 pathway. To date, there was no approved therapy to target TGF-β1 for the treatment of epidural fibrosis and epidural adhesions after laminectomy. Our research proved the anti-fibrosis effects of decorin, which may provide an effective and promising treatment for epidural fibrosis and epidural adhesions.

## Data Availability

The raw data supporting the conclusions of this article will be made available by the authors, without undue reservation.
